# Cross-Protective Potential of a Novel Monoclonal Antibody Directed against Antigenic Site B of the Hemagglutinin of Influenza A Viruses

**DOI:** 10.1371/journal.ppat.1000350

**Published:** 2009-03-20

**Authors:** Reiko Yoshida, Manabu Igarashi, Hiroichi Ozaki, Noriko Kishida, Daisuke Tomabechi, Hiroshi Kida, Kimihito Ito, Ayato Takada

**Affiliations:** 1 Department of Global Epidemiology, Hokkaido University Research Center for Zoonosis Control, Sapporo, Hokkaido, Japan; 2 Department of Veterinary Microbiology, Faculty of Agriculture, Tottori University, Tottori, Japan; 3 Laboratory of Influenza Viruses, Department of Virology III, National Institute of Infectious Diseases, Musashimurayama, Tokyo, Japan; 4 Laboratory of Microbiology, Department of Disease Control, Graduate School of Veterinary Medicine, Hokkaido University, Sapporo, Hokkaido, Japan; Mount Sinai School of Medicine, United States of America

## Abstract

The hemagglutinin (HA) of influenza A viruses has been classified into sixteen distinct subtypes (H1–H16) to date. The HA subtypes of influenza A viruses are principally defined as serotypes determined by neutralization or hemagglutination inhibition tests using polyclonal antisera to the respective HA subtypes, which have little cross-reactivity to the other HA subtypes. Thus, it is generally believed that the neutralizing antibodies are not broadly cross-reactive among HA subtypes. In this study, we generated a novel monoclonal antibody (MAb) specific to HA, designated MAb S139/1, which showed heterosubtypic cross-reactive neutralization and hemagglutination inhibition of influenza A viruses. This MAb was found to have broad reactivity to many other viruses (H1, H2, H3, H5, H9, and H13 subtypes) in enzyme-linked immunosorbent assays. We further found that MAb S139/1 showed neutralization and hemagglutination-inhibition activities against particular strains of H1, H2, H3, and H13 subtypes of influenza A viruses. Mutant viruses that escaped neutralization by MAb S139/1 were selected from the A/Aichi/2/68 (H3N2), A/Adachi/2/57 (H2N2), and A/WSN/33 (H1N1) strains, and sequence analysis of the HA genes of these escape mutants revealed amino acid substitutions at positions 156, 158, and 193 (H3 numbering). A molecular modeling study showed that these amino acids were located on the globular head of the HA and formed a novel conformational epitope adjacent to the receptor-binding domain of HA. Furthermore, passive immunization of mice with MAb S139/1 provided heterosubtypic protection. These results demonstrate that MAb S139/1 binds to a common antigenic site shared among a variety of HA subtypes and neutralizes viral infectivity in vitro and in vivo by affecting viral attachment to cells. The present study supports the notion that cross-reactive antibodies play some roles in heterosubtypic immunity against influenza A virus infection, and underscores the potential therapeutic utility of cross-reactive antibodies against influenza.

## Author Summary

Neutralizing antibodies play a critical role in protection from influenza A virus infection. Most neutralizing antibodies recognize hemagglutinin (HA), which is the major surface glycoprotein of influenza viruses. The HA has been classified into sixteen antigenically distinct subtypes. Since HA subtypes of influenza A viruses are principally defined as serotypes determined by neutralization or hemagglutination inhibition tests using polyclonal antisera to the respective HA subtypes, which have little cross-reactivity to the other HA subtypes, it is generally believed that the neutralizing antibodies are not broadly cross-reactive among HA subtypes. Herein we present a novel cross-neutralizing monoclonal antibody that reacts with a variety of HA subtypes in vitro and provides heterosubtypic protection against influenza A virus infections in mice. We demonstrate that this antibody recognizes a common epitope adjacent to the receptor binding region of HA and inhibits virus binding to the cells. The present study supports the notion that cross-reactive antibodies, as well as cytotoxic T lymphocytes, play some roles in heterosubtypic immunity against influenza A virus infection, and underscores the potential therapeutic utility of cross-reactive monoclonal antibodies for multivalent prophylaxis and treatment against infection with influenza A viruses, including the hypothetical new pandemic influenza viruses.

## Introduction

Neutralizing antibodies play a critical role in protection from influenza virus infection. Most neutralizing antibodies recognize hemagglutinin (HA), which is the major surface glycoprotein of influenza viruses. The HA of influenza A viruses has been classified into sixteen antigenically distinct subtypes (H1–H16) that are maintained in avian and mammalian species in nature [1],[2].

HA is responsible for virus entry into target cells, virus binding to the host receptor, internalization of the virus, and subsequent membrane-fusion events. It is initially synthesized as a precursor polypeptide, HA0, that requires proteolytic cleavage into disulfide-linked HA1 and HA2 before it is functional and virus particles are infectious. The major part of HA1 forms the “globular head” region, which contains the necessary structure for binding to the sialic acid receptors. The “stem” region is mostly formed by HA2, which contains the fusion peptide and membrane anchor domain. It has been recognized that there is considerable amino acid variability (antigenic difference) in the globular head region among HA subtypes, whereas the structure of the stem region is relatively conserved.

The HA antigenic structure of the H3 subtype has been well characterized by using the sequence information on naturally occurring and laboratory-selected antigenic variants [3],[4],[5],[6]. Five different antigenic sites have been identified and mapped mainly on the HA1 globular head region in the three-dimensional structure of the H3 HA molecule [3],[4]. Antigenic sites of H1 [7] and H2 [8] subtypes were then characterized by the identification of amino acid substitutions found in the HA sequences of variants that escaped from neutralization by antibodies. Recently, it was suggested that the structures of antigenic sites of H5 [9],[10] and H9 [11] subtypes were different from those of the H1, H2, and H3 subtypes.

In general, HA subtypes of influenza A viruses are principally defined as serotypes determined by neutralization or hemagglutination inhibition (HI) tests using polyclonal antisera to the respective HA subtypes, which have little cross-reactivity to the other HA subtypes. Furthermore, since the structures of HA antigenic sites vary among not only different subtypes of viruses but also the same subtype, it is generally believed that the neutralizing antibodies are not broadly cross-reactive among HA subtypes. Therefore, studies on cross-reactive HA-specific antibodies to multiple HA subtypes have been limited [12],[13],[14].

Recently, it has been shown that intranasal immunization with inactivated viruses provided heterosubtypic protection in a mouse model, suggesting a role for cross-reactive antibodies in the heterosubtypic immunity against influenza viruses [15],[16],[17]. In this study, we generated a novel cross-neutralizing monoclonal antibody (MAb) that reacts with a variety of HA subtypes by intranasal immunization of mice. This antibody recognizes a common epitope on the globular head region of HA and inhibits virus binding to the sialic acid receptors. The present study suggests the further potential of antibodies in heterosubtypic immunity against influenza A virus infection.

## Results

### Characterization of MAb S139/1 in vitro

**Figure 1:**
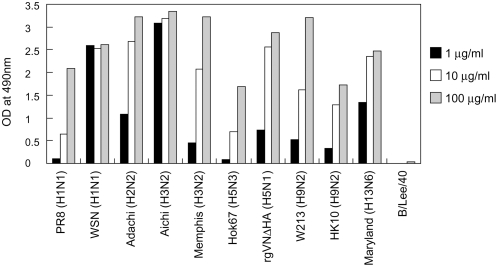
Reactivity of MAb S139/1 to various influenza virus strains. Binding activity of MAb S139/1 at the concentrations of 100 µg/ml (grey), 10 µg/ml (white), and 1 µg/ml (black) to the indicated virus strains was measured by ELISA as described in Materials and Methods.

MAb S139/1 (IgG2a) was originally produced as an H3 HA-specific antibody. The cross-reactivity of MAb S139/1 to multiple subtypes of influenza A virus HAs was then tested by enzyme-linked immunosorbent assay (ELISA) using several H1, H2, H3, H5, H9, and H13 subtypes (Fig. 1). We found that MAb S139/1 reacted with all influenza A virus but not B virus strains tested, with higher binding activities to the A/WSN RG/33 (WSN) (H1), A/Adachi/2/57 (Adachi) (H2), A/Aichi/2/68 (Aichi) (H3), and A/gull/Maryland/704/77 (Maryland) (H13) strains than to the other strains. We confirmed the specificity of MAb S139/1 by Western blotting using purified viruses. MAb S139/1 bound to HA molecules under non-reducing conditions, whereas it bound very weakly to HA1 but not HA2 under reducing conditions (data not shown), suggesting that MAb S139/1 recognized a conformational epitope on the HA1 subunit of the HA molecule.

**Figure 2:**
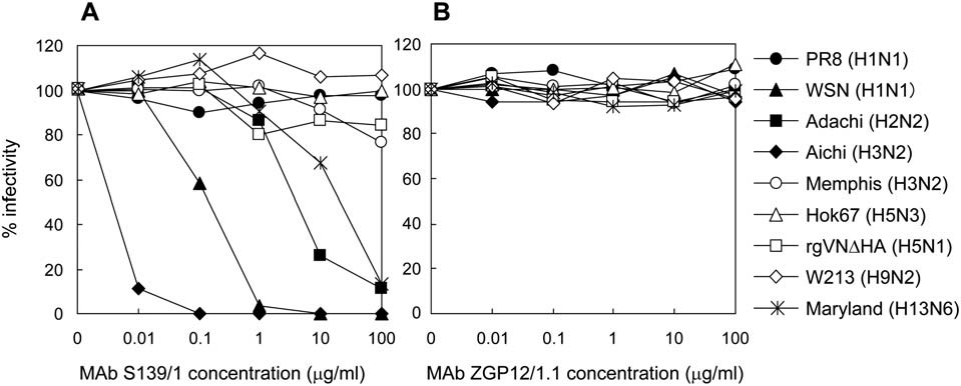
Neutralization activity of MAb S139/1 to various HA subtypes of influenza A virus strains. Viruses were mixed with indicated concentrations of the purified MAb S139/1 (A) or control IgG2a (ZGP12/1.1) [18] (B). Neutralization activities were evaluated by plaque reduction assays using MDCK cells.

We next tested HI activity of MAb S139/1 to various influenza virus strains (Table 1). MAb S139/1 exhibited high HI titers to a particular H1 strain and most of the H3 strains tested, and moderate activity to H2 and H13 strains, but not to H5 and H9 or type B strains. Neutralizing activity of MAb S139/1 was then determined in vitro by a plaque reduction assay using Madin-Darby canine kidney (MDCK) cells (Fig. 2). Consistent with its reactivity profile in HI tests and ELISA, MAb S139/1 neutralized infectivity of WSN (H1), Adachi (H2), Aichi (H3), and Maryland (H13) strains. Relative neutralizing activities of MAb139/1 were also correlated with its reactivity to these viruses in the HI test and ELISA (i.e., MAb S139/1 neutralized Aichi (H3), WSN (H1), Adachi (H2), and Maryland (H13) in order of increasing activity). These results indicated that MAb S139/1 had a novel potential to neutralize the infectivity of multiple subtypes of influenza A viruses by inhibiting HA binding to the sialic acid receptors.

**Table 1:** HI activity of MAb S139/1 with various influenza virus strains.

Virus	HI titer µg/ml a
A/PR/8/34 (H1N1)	>50 b
A/WSN RG/33 (H1N1)	1.56
A/Adachi/2/57 (H2N2)	12.5
A/Singapore/1/57 (H2N2)	6.25
A/duck/Hong Kong/836/80 (H3N1)	<0.39
A/Aichi/2/68 (H3N2)	0.78
A/Memphis/1/96 (H3N2)	>50
A/duck/Hokkaido/5/77 (H3N2)	12.5
A/chicken/Hong Kong/37/78 (H3N2)	<0.39
A/duck/Hokkaido/8/80 (H3N8)	<0.39
A/Hong Kong/483/97 (H5N1)	>50
A/rgViet Nam/1194ΔHA/2004 (H5N1)	>50
A/swan/Hokkaido/67/96 (H5N3)	>50
A/swine/Hong Kong/10/98 (H9N2)	>50
A/duck/Hong Kong/W213/97 (H9N2)	>50
A/duck/Hokkaido/49/98 (H9N2)	>50
A/gull/Maryland/704/77 (H13N6)	12.5
B/Lee/40	>50

a HI titers are expressed as the lowest concentrations of purified MAb S139/1 that completely inhibited hemagglutination.

b No detectable hemagglutination inhibition at 50 µg/ml by MAb S139/1.

### Protective Potential of MAb S139/1 in vivo

**Figure 3:**
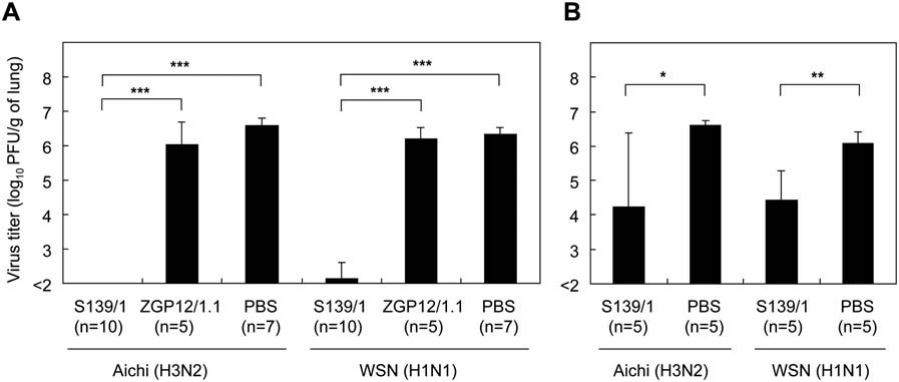
Protective efficacy of passive immunization with MAb S139/1 in mice. Mice were passively immunized before (A) or after (B) virus challenge with Aichi (H3) or WSN (H1). Control mice were given MAb ZGP12/1.1 or PBS. Virus titers of the lung were determined by a plaque assay. The means and standard deviations are shown. For statistical purposes, samples with undetectable virus titers were given the value 2.0 log_10_PFU/g. The data of pre-immunized mice were analyzed using the nonparametric Kruskal-Wallis ANOVA on ranks, followed by the Mann-Whitney U-test with the Bonferroni correction for multiple comparisons. The data of post-immunized mice were analyzed using the Mann-Whitney U-test. Two-sided *p* values less than 0.05 were considered statistically significant. Significant differences were indicated by asterisks (*** *p*<0.001, ** *p*<0.01, * *p*<0.05). All statistical analyses were performed with the computer program R (version 2.8.1).

We then investigated the potential of MAb S139/1 to protect mice against influenza virus infection. Mice were passively immunized by intraperitoneal injection of purified MAb S139/1 one day before or after intranasal challenge with Aichi (H3) or WSN (H1). Control groups were given an irrelevant MAb (ZGP12/1.1) [18] or PBS alone. Protective efficacy was evaluated by titrating infectious virus in the lung tissues three days after challenge (Fig. 3). Mice pre-immunized with MAb S139/1 were almost completely protected from both Aichi (H3) and WSN (H1) infection, while these viruses were recovered from all the control mice at high titers (Fig. 3A). There was no significant difference between two control groups. Post-immunization with MAb S139/1 also significantly reduced both Aichi and WSN titers, as compared with control group (*p*<0.05 and *p*<0.01, respectively) (Fig. 3B). Importantly, virus was not detected from two of the five MAb S139/1-immunized mice infected with Aichi. These results indicate that passive immunization of mice with MAb S139/1 provided heterosubtypic protection against H1 and H3 viruses.

### Amino Acid Substitutions of the Escape Mutants

To determine the epitope for MAb S139/1, escape mutants of Aichi (H3), WSN (H1), and Adachi (H2) were selected in the presence of this antibody as described in Materials and Methods. We confirmed that hemagglutination activities of these escape mutants were not inhibited by MAb S139/1 even at the concentration of 50 µg/ml. The nucleotide sequences of the HA genes of the parent strains and the escape mutants were determined and deduced amino acid sequences were compared among these viruses. We found amino acid substitutions at position 156, 158, or 193 (H3 numbering here and throughout the text) in these mutants (Table 2). Eleven and four escape mutants of WSN (H1) and Adachi (H2), respectively, all acquired the substitution at the same position, 193 (S193N and T193K, respectively). The amino acid residue at position 193 is located on the antigenic sites known as Sb and I-B of H1 [7] and H2 HAs [8], respectively, which correspond to HA antigenic site B of H3 HA [3]. On the other hand, the amino acid substitutions at position 156 (K156Q), 158 (G158E or G158R), or 193 (S193I or S193R) were found in the sixteen escape mutants of Aichi (H3). All these amino acid positions were involved in the conformation of HA antigenic site B [3].

**Table 2:** Amino acid substitutions found in HA of WSN, Adachi, and Aichi escape mutants.

Virus	Amino acid substitution
WSN (H1)	S193N (11/11) a
Adachi (H2)	T193K (4/4)
Aichi (H3)	K156Q (1/16)
	G158E (10/16)
	G158R (1/16)
	S193I (2/16)
	S193R (2/16)

a Total numbers of escape mutants obtained in 2 independent experiments are shown (No. of variants/total escape mutants cloned.)

### Comparison of HA Amino Acid Sequences among Different HA Subtypes

**Figure 4:**
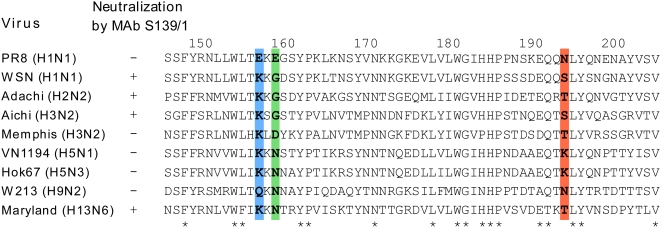
Comparison of the amino acid sequences of different subtypes of influenza A virus HAs. Amino acids at positions from 145 to 204 are shown. Boxed residues indicate the positions 156 (blue), 158 (green), and 193 (red). Asterisks indicate conserved amino acid residues among HA subtypes examined. Amino acid sequences for HAs, except Adachi (H2) and W213 (H9), were downloaded from the Influenza Virus Resource at the National Center for Biotechnology Information (NCBI) (http://www.ncbi.nlm.nih.gov/genomes/FLU/FLU.html) [48]. The NCBI accession numbers are CAA24272 (PR8), AAA43209 (WSN), AB432938 (Adachi), CAA24269 (Aichi), AAB63708 (Memphis), AAT7327 (VN1194), BAE48688 (Hok67), AB432937 (W213), and BAF46906 (Maryland).

We compared deduced amino acid sequences of the region including the MAb S139/1 epitope among H1, H2, H3, H5, H9, and H13 subtypes of HA (Fig. 4). Among these, WSN (H1), Adachi (H2), Aichi (H3), and Maryland (H13), which were neutralized by MAb S139/1, shared the amino acid sequence at positions 156, 158, and 193 (K, G and S/T, respectively), with one exception (N at position 158 in Maryland HA). By contrast, viruses that were not neutralized by MAb S139/1 possessed different sets of amino acids at positions 156, 158, and 193; A/PR/8/34 (PR8) (H1) (E, E and N), A/Memphis/1/96 (Memphis) (H3) (K, D, and T), A/Viet Nam/1194/2004 (VN1194) (H5) (K, N, and K), A/swan/Hokkaido/67/96 (Hok67) (H5) (K, N, and K) and, A/duck/Hong Kong/W213/97 (W213) (H9) (Q, N, and N), respectively.

### Comparison of the Epitope Structure between Aichi and Other Viruses

**Figure 5:**
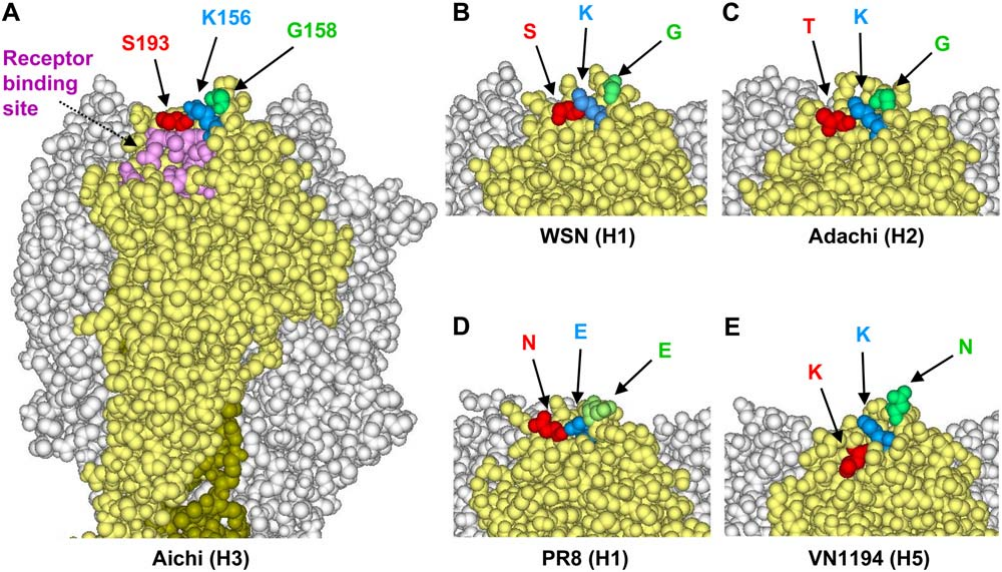
Structure of the MAb S139/1 epitope on the globular head of HA trimer models. Three-dimensional models of Aichi (H3) (A), PR8 (H1) (D), and VN1194 (H5) (E) HAs were constructed from the coordinates obtained from the Protein Data Bank (PDB codes: 1HGF, 1RVX, and 2IBX, respectively). The structures of WSN (H1) (B) and Adachi (H2) (C) were constructed by homology modeling as described in Materials and Methods. Images were prepared by using DS Visualizer (version 1.7, Accelrys, Inc.). Residue numbering is thoroughly on the basis of the H3 HA sequence.

In three-dimensional structural analysis of the Aichi (H3) HA molecule, positions of the amino acid substitutions found in the escape mutants were mapped in the globular head of the HA (Fig. 5A). We found that these three amino acids formed a conformational epitope that was adjacent to the receptor-binding site of the HA. We then compared the structure of this epitope among Aichi (H3), WSN (H1), Adachi (H2), PR8 (H1), and VN1194 (H5) HAs (Fig. 5). The epitopes formed by amino acid residues at positions 156, 158, and 193 were similar among Aichi (H3), WSN (H1), and Adachi (H2), whereas the structures of PR8 (H1) and VN1194 (H5) epitopes were distinct in the amino acid properties or side-chain orientations at positions 156, 158, and 193. A significant difference of amino acid properties at positions 158 and 193 between PR8 (H1) and Aichi (H3) was that the molecular sizes of amino acid residues E and N (PR8) were larger than G and S (Aichi). In VN1194 (H5) HA, a significant difference was found in side chain orientation, which was presumably because of electrostatic repulsion between the positively charged K156 and K193 side chains.

### Neutralization of PR8 Mutants Possessing the Modified Epitope for MAb S139/1

**Figure 6:**
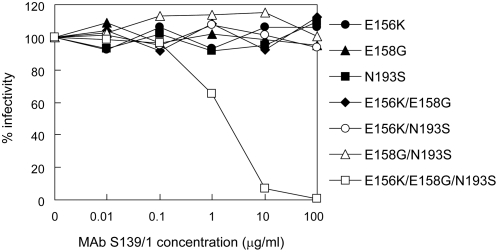
Neutralizing activity of MAb S139/1 to PR8 mutants with altered epitopes. Amino acids at positions 156, 158, and/or 193 of PR8 HA were substituted for by those at corresponding positions of the Aichi HA sequence. Other experimental conditions were described in Materials and Methods.

To confirm the importance of the amino acid residues at positions 156, 158, and 193 for binding of MAb S139/1, we generated recombinant PR8 (H1) mutant viruses with modified epitopes whose amino acid sequences at these positions were replaced with those of Aichi (H3), and tested neutralizing activities of MAb S139/1 to the mutant viruses (Fig. 6). Of the seven mutants generated, three had a single substitution (E156K, E158G, or N193S), three had double substitutions (E156K/E158G, E156K/N193S, or E158G/N193S), and one had triple substitutions (E156K/E158G/N193S). We found that only the triple mutant was neutralized by MAb S139/1 (Fig. 6). Accordingly, MAb S139/1 bound to the triple mutant more efficiently than to the other mutants and parent PR8 in ELISA (data not shown). These results suggest that all three amino acids, K, G, and S at positions 156, 158, and 193, respectively, are equally important components to form this epitope (i.e., MAb S139/1 recognizes this conformational epitope through interaction with all three of these amino acid residues).

## Discussion

It has been generally known that hetrosubtypic immunity can be provided by subtype cross-reactive cytotoxic T lymphocytes that recognize conserved epitopes of viral internal proteins of influenza A viruses such as nucleoprotein and matrix protein [19]. However, recent studies in mouse models suggest that humoral immunity, B cells and antibodies, also contribute to heterosubtypic protection [15],[16],[17],[20]. In the present study, we obtained a novel monoclonal antibody, MAb S139/1, which was broadly cross-reactive to a variety of HA subtypes of influenza A viruses. MAb S139/1 most likely neutralized the viral infectivity by blocking receptor binding of the virus, since hemagglutination of the viruses was also inhibited by this antibody. Influenza virus HA subtypes are determined as serotypes based on their distinct antigenicities, and thus there are a limited number of studies reporting such heterosubtypic and cross-reactive MAb to HA [12],[13],[14],[21],[22].

For example, MAb IVA1B10 [12],[21] and MAb HA1-66 [13],[22], both of which recognize the HA1 region, were shown to react with H3, H4, H11, and H13 strains, but did not have HI and neutralization activities. Some MAbs recognizing the HA2 region were also shown to be cross-reactive among influenza virus strains of the same subtypes, and even among various subtypes [12],[21],[22],[23]. However, these antibodies neither prevented hemagglutination nor neutralized infectivity of the viruses [12],[21],[22],[23]. On the other hand, it was shown that cross-reactive MAb C179, specific to both H1 and H2 subtypes, neutralized viral infectivity [14], though this MAb did not show HI activity. It recognizes a conformational epitope consisting of HA1 and HA2 in the middle of the stem region, a conserved antigenic site among the subtypes, suggesting that inhibition of the low pH-induced conformational change followed by membrane fusion is the mechanism underlying the action of this antibody [14],[24]. By contrast, this study for the first time demonstrates that there is a common epitope shared among multiple HA subtypes, which is recognized by a neutralizing antibody that prevents receptor binding of the virus.

In the present study, we found that MAb S139/1 bound to all the strains of H1, H2, H3, H5, H9, and H13 subtypes tested by ELISA, whereas it showed neutralization and HI activities to some particular strains tested. Since we assume that MAb S139/1 recognizes a single epitope on the HA molecules of all the subtypes, it is likely that the different binding affinity of MAb S139/1 to each HA subtype influences the neutralization and HI activities. Indeed, our data demonstrated that there was an appreciable correlation between its binding affinities tested by ELISA and by HI or the neutralization test. Furthermore, our sequence analyses and reverse genetics approaches revealed the major contribution of the HA amino acid residues at positions 156, 158, and 193 to the binding capacity of MAb139/1.

We found three independent substitutions at positions 156, 158, or 193 in the Aichi mutant viruses that escaped from neutralization by MAb S139/1. Three-dimensional structural analyses revealed a conformational epitope consisting of these amino acid residues. Of these, the amino acid residue at position 193 of H3 HA was shown to interact with the host receptor molecule (i.e., sialic acid-linked oligosaccharide) [5],[25],[26],[27],[28], suggesting the contribution of this residue to receptor binding of the HA. Accordingly, Aichi escape mutants with a substitution at position 193 (S193I and S193R) formed significantly smaller plaques than the Aichi parent virus (data not shown), which might have resulted from the reduced HA function of these mutants.

Three-dimensional structural analyses based on HA molecules cocrystallized with a sialylated glycan receptor analogue (pentasaccharide) suggest that molecular contacts between HA and the sialylated glycan receptor are divided into base and extension regions which include contacts with the terminal sialylgalactose moiety and the subsequent sugar rings, respectively [29],[30], and that the sialylated glycan molecules bind to H1 and H3 HAs in different conformations (i.e., the H1 HA glycan binding site in the extension region form different conformation from that of H3 HA) [27],[29],[30],[31],[32],[33]. Since the amino acid residue at position 193 of H3 HA seems to directly interact with a specific sugar ring in the extension region [27],[31],[33], mutation at this position of the H3 HA likely influence its receptor binding properties. Consistent with this hypothesis, an Aichi (H3) escape mutant (S193I) had reduced ability to grow in cell culture and in mice (data not shown). On the other hand, it has been suggested that the amino acid residues not only at position 193 but also at position 190 of H1 HA play a key role in interaction with the glycan in the extension region [27],[29],[31],[32]. Since the amino acid substitution at position 190 of H1 HA is believed to be responsible for the alteration of receptor binding properties [29],[31], a single mutation at position 193 found in WSN (H1) escape mutants might have a limited effect on the overall receptor binding capacity of the HA.

It is well-known that HAs of avian and human influenza viruses bind preferentially to α2-3 and α2-6 sialylated (SA) glycan receptors, respectively. Although it has been generally believed that amino acid substitutions at positions 226 and 228 are primarily responsible for the differences in the receptor specificity between avian and human H3 viruses, several studies have reported that the mutation at position 193 of H3 HA might also alter the receptor binding properties [5],[31],[33],[34]. For example, S193R substitution on prototype H3 human virus HA altered binding specificity by acquiring the ability to agglutinate erythrocytes containing SAα2-3Gal linkage [5], while a single S193R substitution of H3 HA in a recent human virus enhanced SAα2-6Gal but not SAα2-3Gal recognition [34]. Thus, it is conceivable that H3 escape mutants that are naturally selected by an antibody recognizing the S139 epitope may have reduced receptor binding capacity and/or altered receptor specificity. Moreover, amino acid positions 155, 159, 190, and 225 (H3 numbering) of H1 HA, most of which cluster around this epitope, have also been demonstrated to influence receptor specificity [31]. Thus, it might be of interest to clarify whether the reactivity of MAb S139/1 to multiple HA suptypes is affected by changes of HA receptor recognition associated with substitutions around this epitope.

Recently, passive transfer of MAbs specific to viral proteins has been tested in clinical studies, providing models for the use of MAbs for prophylaxis or treatment of infectious diseases. In fact, a humanized MAb specific to RSV F protein is already approved by the US Food and Drug Administration and used in clinical cases. It has been experimentally shown that this approach is effective for influenza virus infection in mice [24],[35],[36],[37]. The present study further indicated that the MAb S139/1 provided heterosubtypic protection of mice from H1 and H3 influenza A virus infection. It may be one of the options in the event of pandemic influenza [36]. Although MAb S139/1 neutralizes only particular strains of H1, H2, H3, and H13 subtypes, this antibody has binding capacity to other virus strains of different subtypes (Fig. 1). Since in vitro neutralization activity was not necessarily linked to the protective potential in vivo (e.g., non-neutralizing MAbs such as anti-HA2 MAbs [22],[37] and anti-M2 MAbs [38],[39],[40] protected mice from lethal influenza A virus infection), it may be interesting to estimate the broader heterosubtypic protective efficacy of passive transfer of MAb S139/1 in animal models.

Together with previous studies by others [22],[24],[36],[37],[38],[39],[40], the present study supports the notion that cross-reactive antibodies, as well as cytotoxic T lymphocytes, play some roles in heterosubtypic immunity against influenza A virus infection, and underscores the potential therapeutic utility of cross-reactive MAbs for multivalent prophylaxis and treatment against infection with influenza A viruses, including the hypothetical new pandemic influenza viruses.

## Materials and Methods

### Viruses and Cells

Influenza virus strains, A/PR/8/34 (PR8) (H1N1), A/WSN RG/33 (WSN) (H1N1), A/Adachi/2/57 (Adachi) (H2N2), A/Singapore/1/57 (H2N2), A/duck/Hong Kong/836/80 (H3N1), A/Aichi/2/68 (Aichi) (H3N2), A/Memphis/1/96 (Memphis) (H3N2), A/duck/Hokkaido/5/77 (H3N2), A/chicken/Hong Kong/37/78 (H3N2), A/duck/Hokkaido/8/80 (H3N8), A/Hong Kong/483/97 (H5N1), A/rgViet Nam/1194ΔHA/2004 (rgVNΔHA) (H5N1), A/swan/Hokkaido/67/96 (Hok67) (H5N3), A/swine/Hong Kong/10/98 (HK10) (H9N2), A/duck/Hong Kong/W213/97 (W213) (H9N2), A/duck/Hokkaido/49/98 (H9N2), A/gull/Maryland/704/77 (Maryland) (H13N6), and B/Lee/40 were used (Table 1). rgVNΔHA (H5N1) is a reassortant virus that has the modified H5 HA gene derived from A/Viet Nam/1194/2004 (VN1194) (H5N1) and all other genes from PR8. In this modified H5 HA, the original amino acid sequences at the cleavage site (PQRERRRKKRG) were replaced with those of A/teal/Hong Kong/W312/97 (H6N1) (PQIETRG). All infectious materials were handled in a biosafety level 2 or 3 facility under approved protocols in accord with guidelines of Hokkaido University. These viruses, except a highly pathogenic virus, A/Hong Kong/483/97, were propagated in the allantoic cavities of 10-day-old embryonated chicken eggs at 35°C for 48 h (A/Hong Kong/483/97 was incubated in eggs for 36 hours). Some of these viruses were concentrated and purified by high-speed centrifugation of infected allantoic fluid passed through a 10 to 50% sucrose density gradient [16]. The purified viruses were resuspended in phosphate-buffered saline (PBS) and stored at −80°C until use. Madin-Darby canine kidney (MDCK) cells were maintained in Eagle's minimal essential medium (MEM) supplemented with 10% calf serum. Human embryonic kidney 293T cells were maintained in Dulbecco's modified Eagle's medium supplemented with 10% fetal calf serum.

### Monoclonal Antibodies (MAbs)

Six-week-old female BALB/c mice, were intranasally immunized twice at 2-week intervals with 100 µg of formalin (0.2%)-inactivated purified Aichi together with cholera toxin B (Sigma). Two weeks after the second immunization, the mice were intranasally boosted with inactivated virus alone. Three days later, the spleen cells from the mice and mouse myeloma Sp2/0 cells were fused and maintained according to a standard procedure [41]. Hybridomas were screened for secretion of anti-influenza-virus specific MAbs by enzyme-linked immunosorbent assay (ELISA), and then HA-specific MAbs were identified by Western blotting and immunostaining of 293T cells transfected with plasmids expressing Aichi HA. We further screened for cross-reactivity of the antibodies to other HA subtypes by ELISA, and obtained cross-reactive MAb S139/1 (IgG2a). The hybridoma producing MAb S139/1 (IgG2a) was cloned twice by limiting dilution of the cells. MAb S139/1 was purified from mouse ascites using protein A agarose columns (Bio-Rad).

### Antibody Assays

ELISA was performed essentially as previously described [41]. Briefly, purified viruses were disrupted with 50 mM Tris–HCl (pH 7.8) containing 0.5% Triton X-100 and 0.6 M KCl, diluted by PBS, and used for antigen coating (20 µg protein/ml in PBS, 50 µl/well), followed by blocking with BSA. Binding of MAb S139/1 was detected by using peroxidase-conjugated goat anti-mouse IgG (H+L) (Jackson) and *o*-phenylendiamine dihydrochloride (Sigma). HI activity of the purified MAb was tested by the standard method using 0.5% chicken erythrocytes. Neutralizing activity of the MAb was measured by a plaque reduction assay using MDCK cells. Ten-fold dilutions of MAb were mixed with 100–200 plaque forming unit (PFU) of viruses and incubated for 1 h at room temperature. The confluent monolayers of MDCK cells on 12-well plates were inoculated with the mixture. After 1 h adsorption, the virus inoculums were removed and the cells were overlaid with MEM containing 1% Bact-agar and trypsin (5 µg/ml). The plaques were enumerated after incubation at 35°C, 5% CO_2_ for 2 days. Western blotting was performed as follows. Virus proteins were separated by 10% SDS-polyacrylamide gel electrophoresis under reducing or non-reducing conditions, and transferred to PVDF membranes (Millipore). The membranes were blocked with 3% skim-milk (SM) in PBS containing 0.05% Tween-20 (PBST) and exposed to MAb S139/1 (1 µg/ml) in 1% SM-PBST, and then probed with horseradish peroxidase-conjugated goat anti-mouse IgG (H+L) (Jackson), and the reacted bands were visualized by Immunostaining HRP-1000 (Konica Minolta).

### Passive Immunization and Protection Tests

The experimental protocols were reviewed and approved by the Hokkaido University Animal Care and Use Committee (08-0234). Six-week-old female BALB/c mice were passively immunized by intraperitoneal injection with 200 µg of purified MAb S139/1 or ZGP12/1.1 (IgG2a) [18] in 0.5 ml of PBS. Twenty four hours before or after immunization, mice were challenged intranasally with 50 µl of 10×50% mouse infectious dose of Aichi (H3) or WSN (H1) under anesthesia with isoflurane. Three days after the challenge, mice were euthanized to obtain the lung tissue samples. The lung homogenates (10% w/v) prepared in MEM were disrupted and centrifuged at 3,000×g for 10 min, and then the supernatants were examined for virus infectivity. Virus titers were measured by a plaque assay using MDCK cells.

### Sequence Analysis of the HA Genes

Viral RNA was extracted using a QIAamp Viral RNA Mini Kit (Qiagen). After reverse transcription with M-MLV reverse transcriptase (Invitrogen) using Uni12 primer (5′-AGCAAAAGCAGG), HA genes were amplified by PCR using gene-specific primer sets [42]. PCR products were purified with a QIAQuick PCR purification kit (Qiagen) and nucleotide sequences were analyzed using a dye-terminator cycle sequencing system with an ABI sequencer (Perkin-Elmer, Applied Biosystems).

### Selection of Escape Mutants

Escape mutants were selected by culturing WSN (H1), Adachi (H2), and Aichi (H3) strains in MDCK cells in the presence of MAb S139/1. Viruses were incubated with purified MAb S139/1 (final concentration of 10 µg/ml) for 1 h, and then the mixtures were inoculated into confluent MDCK cells in 6-well tissue culture plates. After 1 h adsorption, the cells were overlaid with MEM containing 1% Bacto-Agar (Difco) and MAb S139/1 ascites (final dilution of 1∶200) and trypsin (5 µg/ml), and then incubated for 2 days at 35°C. Escape mutants were purified from single isolated plaques, and propagated in MDCK cells with serum-free MEM containing trypsin. The nucleotide sequences of the HA genes of the parent strains and the escape mutants were determined and deduced amino acid sequences were compared among these viruses.

### Generation of Recombinant Mutant Viruses by Reverse Genetics

The plasmid pWH194-HA [37] expressing PR8 HA was modified using a QuickChange II Site-Directed Mutagenesis kit (Stratagene). HA mutant viruses were generated by the reverse genetics method as described previously [43]. Briefly, 293T and MDCK cells were cocultured on 6-well plate and transfected with a set of eight influenza virus plasmids allowing the rescue of the recombinant PR8 (H1N1) for generating all HA mutants. The recombinant viruses produced from transfection were amplified in MDCK cells, and stored at −80°C. The HA genes of the recombinant viruses were sequenced to verify the presence of the desired mutations and the absence of other changes.

### Molecular Modeling

The HA structures of WSN (H1) and Adachi (H2) were constructed using Modeller 8v2 [44] based on the crystal structures of H1 (PDB code: 1RU7) and H5 (PDB code: 2FK0) HA molecules, respectively. After one hundred models of the HA trimer were generated, we selected the model with the best probability distribution function (PDF) score. The HA model was evaluated by using PROCHECK [45], WHATCHECK [46], and VERIFY-3D [47].
